# EndoArt in Corneal Endothelial Disease: Systematic Review and Meta-Analysis of Clinical Outcomes and Complications

**DOI:** 10.7759/cureus.100619

**Published:** 2026-01-02

**Authors:** Muneeb A Khan, Diya Baker, Mohammad Karam, Afaq Khan, Narvair Kahlar, Abdulmalik Alsaif

**Affiliations:** 1 General Practice, Royal Stoke University Hospital, Stoke-on-Trent, GBR; 2 Ophthalmology, Royal Wolverhampton NHS Trust, Wolverhampton, GBR; 3 Ophthalmology and Visual Sciences, McGill University, Montreal, CAN; 4 Otolaryngology, Scunthorpe General Hospital, Scunthorpe, GBR; 5 Ophthalmology, Birmingham and Midland Eye Centre, Birmingham, GBR; 6 Ophthalmology, King Khaled Eye Specialist Hospital, Riyadh, SAU

**Keywords:** artificial corneal endothelial implant, chronic corneal oedema, corneal endothelial failure, dmek, dsaek, endoart

## Abstract

The purpose of this study was to systematically evaluate the safety, clinical outcomes, and complications of EndoArt (EyeYon Medical, Nes Ziona, Israel) as an alternative to donor endothelial keratoplasty for chronic corneal oedema.

MEDLINE/EMBASE, PubMed, Cochrane Library, and Google Scholar were searched for studies published between 1st Jan 2020 and 17th May 2025 reporting clinical outcomes following EndoArt implantation. A total of 12 studies were included. Eligible studies included case reports, case series, retrospective observational studies, and single-arm interventional studies. Overlapping cohorts were resolved by retaining the most complete datasets. Risk of bias was assessed by the Joanna Briggs Institute critical appraisal tools and the Methodological Index for Non-randomised Studies. Quantitative synthesis was pre-specified for outcomes with ≥2 studies; meta-analysis used standardised mean differences or pooled event rates with heterogeneity.

Four studies (56 eyes) were included for quantitative analysis. Pooled visual acuity improved (SMD −0.728; 95% CI −1.277 to −0.180; p=0.009); mean visual acuity changed from 1.561 to 1.212 logMAR with low heterogeneity (I² = 20.428%, p = 0.285). Central corneal thickness decreased (SMD −1.420; 95% CI −1.867 to −0.974; p<0.001); mean central corneal thickness fell from 755.46 to 554.07 µm with low heterogeneity (I² = 0%). The pooled re-bubbling rate was 60.2% (95% CI 38.7 to 78.4%; I² = 52.88%). The most frequently reported adverse events included partial implant detachment and raised intraocular pressure.

EndoArt achieves meaningful deturgescence and visual acuity gains, but at the cost of high detachment and rebubbling rates. Given current evidence, EndoArt is likely best suited as salvage therapy, offering independence from donor tissue. No immune response was reported. Larger cohort comparative studies are required to refine indications and technique.

## Introduction and background

Corneal endothelial failure, most commonly from Fuchs endothelial corneal dystrophy, pseudophakic bullous keratopathy, or failed endothelial keratoplasty, produces chronic stromal oedema, reduced vision, pain, and marked impairment of quality of life. Endothelial keratoplasty (EK), in particular Descemet membrane endothelial keratoplasty (DMEK) and Descemet stripping automated endothelial keratoplasty (DSAEK), is the current standard of care and can restore meaningful visual function in otherwise healthy eyes [1,2]. However, EK depends on viable donor tissue, can be complicated by graft detachment and immunologic rejection, and achieves poorer results in eyes with complex anterior-segment pathology or multiple prior surgeries [3].

EndoArt is a permanently implanted, dome-shaped posterior lamellar device composed of a biocompatible hydrophilic acrylic copolymer that functions as an inert physical barrier to aqueous ingress at the posterior stroma [4]. The device is manufactured as an optically clear, dome-shaped plate designed to match posterior corneal curvature with a diameter and curvature radius of 6.0 - 8.0 mm and a thickness of 30 - 50 μm [4]. The implant is reported to have a three-year shelf life and is intended to be used as an endothelial prosthesis in patients with chronic corneal oedema secondary to endothelial dysfunction [4]. EndoArt received CE (European Conformity) marking for clinical use in June 2021 and is certified under the EU’s Medical Device Regulation (MDR).

Early clinical series since 2020 report rapid stromal deturgescence in many treated eyes, but also relatively high rates of implant dislocation and wide variation in insertion technique [5-8]. Because EndoArt functions through a fundamentally different mechanism than EK, key uncertainties remain regarding the magnitude and durability of its visual and anatomic benefits, the incidence and clinical impact of device detachment, and the surgical approaches most likely to ensure long-term stability. However, the existing literature consists largely of very small cohorts with heterogeneous techniques, inconsistent outcome definitions, and limited or variable follow-up, making it difficult to reliably estimate event rates such as detachment or rebubbling or to define expected visual and anatomical outcomes. A systematic review with meta-analysis was therefore undertaken to consolidate available data, improve the precision of pooled estimates, and contextualise sources of heterogeneity.

## Review

Methods

Eligibility Criteria

Studies were deemed eligible if they reported clinical outcomes following implantation of the EndoArt artificial endothelial layer for the management of corneal endothelial decompensation. Eligible study designs included prospective or retrospective case series, case reports, and interventional trials involving human subjects. No restrictions were placed on patient age, geographic setting, aetiology of endothelial dysfunction, or prior surgical history. Only studies published between 1st January 2020 and 17th May 2025 were included.

Where overlapping cohorts were published in multiple reports, the most recent and methodologically comprehensive study was retained. In particular, two were included as new studies despite previous reports having appeared in the prior review by Romano et al. (2024) [9]. The earlier reports that were excluded are Fontana et al. (2024) [10] and Wiedemann et al. (2024) [11]. The earlier reports were excluded, as the updated studies (Fontana et al. [2025], Wiedemann et al. [2024]) [5,7] provided extended follow-up data or included two additional cases each, thus representing the more complete datasets for synthesis.

The total number of cases reported in Lapid-Gortzak et al. (2025) [12] is 10; however, only three cases from this report were included in our review, as the first seven cases overlapped with the report by Daphna et al. (2025) [8]. This was confirmed with the corresponding author.

Preclinical, animal, and purely technical or materials science studies were excluded from the review.

Search Strategy

A literature search was conducted to identify peer-reviewed studies reporting clinical outcomes following EndoArt implantation for corneal endothelial dysfunction. The following databases and platforms were searched: PubMed, Google Scholar, OVID (MEDLINE and EMBASE), and the Cochrane Library. All searches were completed by 17th May 2025. Search terms included “EndoArt” OR “artificial endothelial layer” OR “artificial corneal endothelium”. No filters were applied for publication status or geographic region. The search was limited to studies published from 1st January 2020 to 17th May 2025. Reference lists of included studies and relevant review articles were manually screened to identify additional eligible reports. This review was not registered with any review registry. Study selection was performed by two reviewers. Duplicates were removed, and studies unrelated to ophthalmology or corneal transplantation were excluded during title and abstract screening.

The search yielded 120 records, with 119 from databases and one from reference lists, of which 24 duplicates were removed, resulting in 96 records screened. Of these, 16 reports were sought for retrieval, and 15 full-text articles were assessed for eligibility. A total of 12 studies have been included in this review.

**Figure 1:**
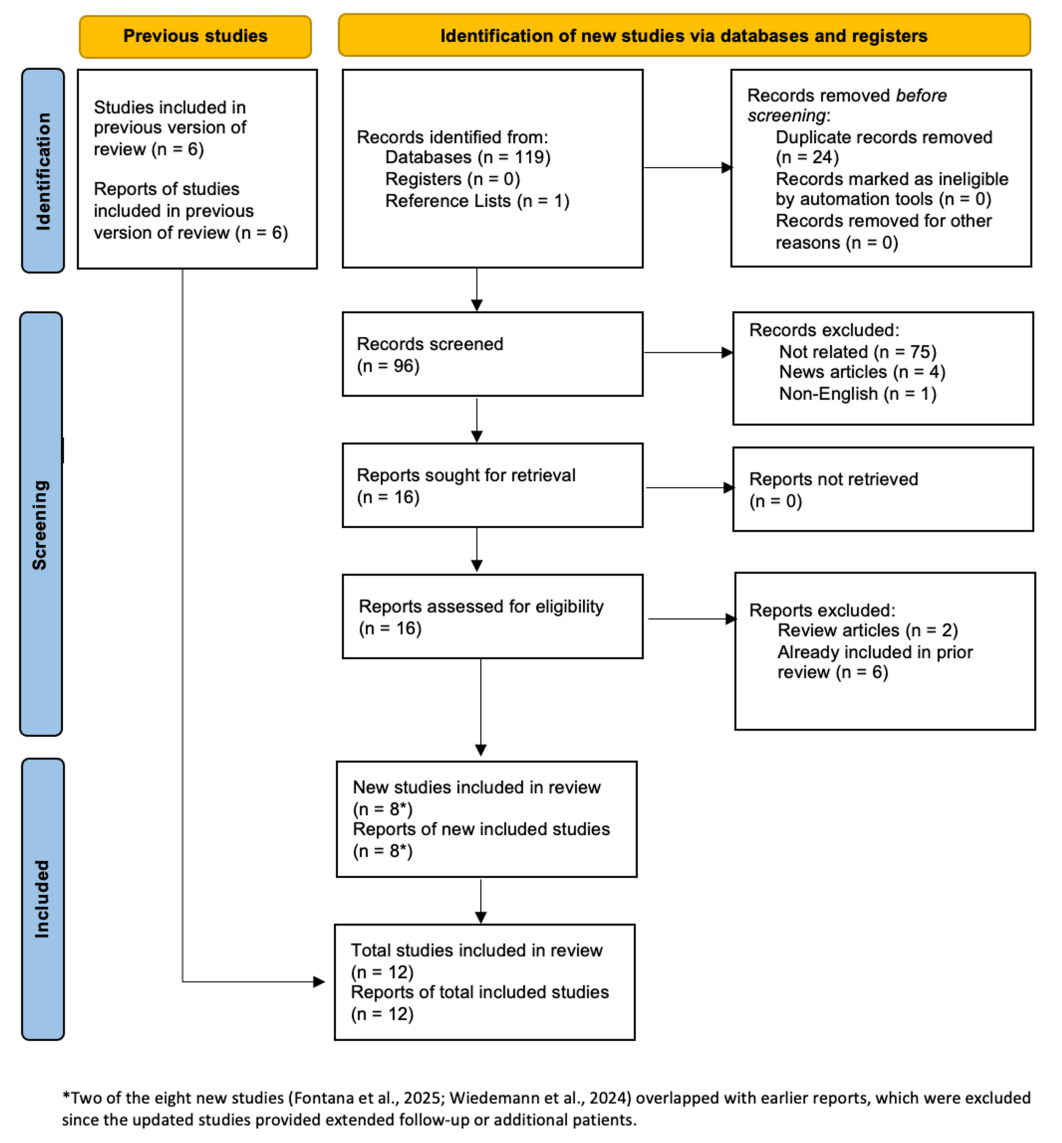
PRISMA flow diagram of study selection PRISMA: Preferred Reporting Items for Systematic Reviews and Meta-Analyses

The study selection process was retrieved from and documented using the PRISMA 2020 flow diagram (Figure 1), and all screening and inclusion criteria decisions were conducted in accordance with PRISMA guidelines.

Data Extraction

Data was collected by two reviewers. For each study, the following were recorded: study name, location, study design, number of eyes, sex, mean age, lens status, ocular co-morbidities, previous ocular surgery, indication for EndoArt, mean follow-up, surgical technique, tamponade used, mean number of anchoring sutures, mean pre-operative VA, mean final VA, mean pre-operative CCT, mean final CCT, eyes with ≥ 1 rebubbling procedures, total rebubbling procedures, and other complications.

Risk of Bias Assessment

Risk of bias was assessed according to design: JBI (Joanna Briggs Institute) Critical Appraisal Tools for case reports [13] and case series [14]; MINORS (Methodological Index for Non-randomised Studies) non-comparative [15] for single-arm interventional study and retrospective observational study.

The results of the risk of bias assessment for case reports and case series are contained in Tables 1, 2, respectively. The risk of bias results for the single-arm interventional study and retrospective observational study are shown in Table 3.

**Table 1:** JBI checklist for case reports Overall, the included case reports demonstrate a low risk of bias, with only isolated unclear items. JBI: Joanna Briggs Institute; N: no; Y: yes; UN: unclear

	Abusayf et al. 2023 [16]	Rens et al. 2024 [17]	Reddy et al. 2024 [18]	Vishwakarma et al. 2024 [19]	Kobayashi et al. 2024 [20]
Patient demographics clearly described?	Y	Y	Y	Y	Y
Patient history clearly described in a timeline?	Y	Y	Y	Y	Y
Clinical condition of patient on presentation clearly described?	Y	Y	Y	Y	Y
Diagnostic/assessment methods and results clearly described?	Y	Y	Y	Y	Y
Intervention/procedure clearly described?	Y	Y	Y	Y	Y
Post-intervention clinical condition clearly described?	Y	Y	Y	Y	Y
Adverse events identified and described?	UN	Y	UN	UN	Y
Takeaway lessons provided?	Y	Y	Y	Y	Y

**Table 2:** JBI checklist for case series The included case series demonstrates a moderate risk of bias due to inconsistent reporting of participant inclusion methods and occasional unclear or incomplete reporting across several domains. JBI: Joanna Briggs Institute; N: no; Y: yes; UN: unclear; N/A: not applicable

	Auffarth et al. 2021 [6]	Romano et al. 2024 [9]	Lapid-Gortzak et al. 2025 [12]	Donner et al. 2025 [21]	Fontana et al. 2025 [5]
Clear criteria for inclusion?	Y	UN	Y	UN	Y
Condition measured in standard, reliable way?	Y	Y	Y	Y	Y
Valid methods used for condition identification?	Y	Y	Y	Y	Y
Consecutive inclusion of participants?	N	UN	N	UN	Y
Complete inclusion of participants?	UN	UN	UN	UN	UN
Clear reporting of participant demographics?	Y	Y	N	N	Y
Clear reporting of participant clinical information?	Y	Y	Y	Y	Y
Outcomes or follow-up results reported?	Y	Y	Y	Y	Y
Clear reporting of demographic information of presenting site?	Y	UN	Y	Y	Y
Appropriate statistical analysis?	N/A	N/A	Y	UN	Y

**Table 3:** MINORS tool (non-comparative) Both studies demonstrated moderate methodological quality, with total MINORS scores of 10/16 and 12/16. These scores reflect several unmet criteria. Most notably, lack of prospective design, absence of prospective sample size calculation, and inconsistent consecutive enrolment. MINORS: Methodological index for non-randomised studies

	Wiedemann et al. 2024 [7]	Daphna et al. 2025 [8]
A clearly stated aim	2	2
Inclusion of consecutive patients	2	1
Prospective collection of data	0	2
Endpoints appropriate to the aim of the study	2	2
Unbiased assessment of the study endpoint	1	1
Follow-up period appropriate to the aim of the study	2	2
Loss to follow-up less than 5%	1	2
Prospective calculation of the study size	0	0
Total	10/16	12/16

Synthesis Methods

Analyses were conducted using Comprehensive Meta-analysis (CMA) version 4.0 (Biostat, Englewood, NJ, USA) using pooled study-level data, with each unit being an individual eye undergoing EndoArt implantation. Pooled event rates with corresponding 95% confidence intervals (CIs) were calculated for dichotomous variables. Standardised mean differences (SMDs) with associated 95% CIs were reserved for continuous outcomes. We predefined a minimum sample size of ≥ 5 eyes per study for inclusion in quantitative meta-analysis to limit small-study effects from isolated case reports or small case series. Each outcome was included in quantitative analysis if at least two studies provided quantitative data; otherwise, outcomes were described qualitatively.

The meta-analysis was conducted using the DerSimonian and Laird model, and weights were calculated using the inverse variance method [22]. The threshold for type I error for statistical significance was set as 𝛼 = 0.05. Between-study heterogeneity was evaluated using Cochran’s Q test and was quantified by the I^2^ statistic, with values of 25% to 49%, 50% to 75%, and 75% or greater being categorised as low, moderate, and high heterogeneity [23].

Results

A total of 12 studies reporting outcomes for 68 eyes undergoing EndoArt implantation were included in this review. To avoid unstable variance estimates from extremely small case series or case reports, only studies including five or more eyes were included in the meta-analysis. Four studies involving 56 eyes provided data suitable for quantitative synthesis. Across the pooled dataset, EndoArt implantation resulted in a mean reduction in CCT from 755.46 μm preoperatively to 554.07 μm postoperatively, corresponding to a significant pooled standardised mean difference of -1.420 (95% CI -1.867 to -0.974). Mean VA improved from 1.561 logMAR to 1.212 logMAR, with a pooled SMD of -0.728 (95% CI -1.277 to -0.180). The most frequently reported complications were implant detachment and rebubbling, with a pooled rebubbling rate of 60.2%. Additional complications included raised intraocular pressure, ocular pain, conjunctivitis, implant explantation due to inadequate attachment, subepithelial fibrosis, and microbial keratitis.

Study Characteristics

Table 4 lists the study characteristics of included studies.

**Table 4:** Study characteristics *seven patients were excluded due to overlapping cohorts from a previous study. DMEK: Descemet membrane endothelial keratoplasty; PPV: Pars plana vitrectomy; GDI: Glaucoma drainage implant; RD: Retinal detachment; SF IOL: Scleral-fixated intraocular lens; AS: Anterior segment; PK: Penetrating keratoplasty; DSAEK: Descemet stripping automated endothelial keratoplasty; RTA: Road traffic accident; DSO: Descemet stripping only; PBK: Pseudophakic bullous keratopathy; CMO: Cystoid macular oedema; CNV: Choroidal neovascularisation

Study	Location	Study Design	Eyes (n)	Sex M/F (%)	Age (Mean +- SD)	Lens Status	Ocular Co-morbidities	Previous Corneal Surgery	Indication for EndoArt
Auffarth et al. 2021 [6]	Germany	Case Series	2	50/50	70 +- 16.97	pseudophakia (n=1)	-	DMEK, PPV for endophthalmitis (n=1), DMEK (n=1)	Pseudophakic bullous keratopathy (n=1), Fuchs Endothelial Dystrophy (n=1)
Abusayf et al. 2023 [16]	Singapore	Case Report	1	100/0	48	pseudophakia	traumatic aphakic glaucoma, GDI with revisions x3, PPV for RD, SF IOL	transcleral cyclophotocoagulation, glaucoma drainage implants X2, PPV for RD	Pseudophakic bullous keratopathy
Rens et al. 2024 [17]	Belgium	Case Report	1	0/100	81	pseudophakia	AS with Anterior Uveitis, complicated PK, Glaucoma	PK X2, DSAEK	Pseudophakic bullous keratopathy
Reddy et al. 2024 [18]	India	Case Report	1	0/100	56	-	macular corneal dystrophy variant with endothelial decompensation	-	macular corneal dystrophy with endothelial decompensation
Wiedemann et al. 2024 [7]	Germany	Retrospective Observational Study	14	42.9/57.1	63.6 +- 17.3	pseudophakia (n=11), aphakia (n=2), phakia (n=1)	chronic glaucoma (n=14), aphakic glaucoma (n=2), anterior synechiae (n=2), aniridia (n=1), iris implant (n=1), recurrent uveitis (n=4), herpetic uveitis (n=3), Fuchs Dystrophy (n=3), Corneal ulcer history (n=1), pseudoexfoliation (n=1), Keratoconus (n=1)	Glaucoma Surgeries (n=11) with GDI (n=7), DMEK (n=10), PK (n=2)	post-op corneal decompensation (n=10), pseudophakic/aphakic bullous keratopathy (n=3), Other (n=1)
Vishwakarma et al. 2024 [19]	Germany	Case Report	1	0/100	59	pseudophakia	RTA with open globe injury and corneal perforation, secondary glaucoma	cyclophotocoagulation, iris diaphragm and SF-IOL, DMEK x3	Pseudophakic bullous keratopathy with endothelial failure and and chronic corneal oedema
Kobayashi et al. 2024 [20]	Japan	Case Report	1	0/100	82	pseudophakia	Fuchs Corneal Endothelial Dystrophy, Band Keratopathy, epiretinal membrane	DMEK	DMEK Failure for Fuchs Corneal Endothelial Dystrophy
Romano et al. 2024 [9]	Italy	Case Series	2	50/50	71 +- 5.66	pseudophakia (n=1)	Corneal perforation due to microbial keratitis (n=1)	Failed DSAEK X2, PK (n=1), Failed PK X3 (n=1)	Failed DSAEK X2 and failed PK (n=1), Failed PK X3, failed DSAEK (n=1)
Daphna et al. 2025 [8]	Multicentre	Multicentre, non-randomised, single-arm Intervention	24	58.3/41.7	69.8 +- 9.6	pseudophakia (n=24)	Glaucoma (n=4), Filtering Surgery (n=2), RD (n=2), History of RD (n=3), vitreal or retinal disease (n=5)	DMEK (n=2), DSAEK (n=2), DSO (n=1)	chronic corneal oedema
Lapid-Gortzak and Van Der Meulen 2025 [12]	Netherlands	Case Series	3*	N/A	71.33 +- 14.64	pseudophakia (n=1)	Keratoconus, GDI, irregular surface of post-PK (n=1) PBK, chronic CMO, History of Uveitis (n=1) History of penetrating eye trauma, sulcus IOL, limbal stem cell deficiency (n=1)	PK, DSAEK, DMEK (n=1), DSAEK (n=1), PK with fibrotic edges (n=1)	Keratoplasty failure (n=1), painful bullous keratopathy (n=2)
Donner et al. 2025 [21]	Austria	Case Series	11	N/A	-	-	Glaucoma (n=8), GDI (n=2), trabeculectomy (n=3), CMO (n=3), Uveitis (n=2), PAS (n=3), CNV (n=2), History of chemical eye injury (n=2), Trauma (n=1), iris implant (n=1), AC IOL (n=1), RD (n=1), Cyclodialysis (n=1), limbal stem cell deficiency (n=1), vitreous extraction and membrane peel (n=1)	DMEK (n=7), PK (n=3)	Endothelial dysfuntion from previous surgery
Fontana et al. 2025 [5]	Italy	Case Series	7	42.9/57.1	76 +- 4	pseudophakia (n=7)	Glaucoma (n=5), history of filtering surgery (n=4),	DSAEK (n=4), DMEK (n=1)	endothelial keratoplasty failure (n=5), bullous keratopathy (n=2)

Outcomes of EndoArt

Table 5 lists the outcomes of EndoArt for the 12 included studies.

**Table 5:** Outcomes of EndoArt *seven patients were excluded due to overlapping cohorts from the previous study. †one patient improved from counting fingers to 0.5 logMAR, two showed no significant change. VA: Visual acuity; logMAR: logarithm of the minimum angle of resolution; CCT: Central corneal thickness; DMEK: Descemet membrane endothelial keratoplasty; C3F8: Perfluoropropane gas; CF: Counting fingers; SF6: Sulphur hexafluoride gas; HM: Hand movements; N/A: Not applicable

Study	Eyes (n)	Mean Follow-up (months)	Surgical Technique	Tamponade Used	Mean number of Anchoring Sutures	Mean Pre-op VA (logMAR)	Mean Final VA (logMAR)	Mean Pre-op CCT (um)	Mean Final CCT (um)	reduction in mean CCT (%)
Auffarth et al. 2021 [6]	2	17	push-through technique with blunt tipped spatula	-	-	1.5 +- 0.57	1.35 +- 0.49	745.5 +- 21.92	491.5 +- 48.79	34.1%
Abusayf et al. 2023 [16]	1	12	pull-through insertion with curved DMEK forceps	12% C3F8	3	CF	1.3	911	691	24.1%
Rens et al. 2024 [17]	1	12	push-through technique using a lens spatula	10% C3F8 and air	1	CF	0.13	1005	661	34.2%
Reddy et al. 2024 [18]	1	15	modified tuck-in via pull-in technique	air	0	1.78	0.3	793	509	35.8%
Wiedemann et al. 2024 [7]	14	2.4	inserted with blunt spatula and centered with price hook	20% SF6 (n=3), 12% C3F8 (n=11)	2	1.6 (+- 0.6)	1.4 +- 0.6	771.8 +- 157	583.2 +- 169.8	24.4%
Vishwakarma et al. 2024 [19]	1	1.5	pull-through insertion with 10-0 prolene suture pulling and pushing implant with spatula	12% C3F8	3	1.22	0.8	1062	751	29.3%
Kobayashi et al. 2024 [20]	1	3	Busin glide-assisted pull-through insertion	20% SF6	1	HM	2	845	530	37.3%
Romano et al. 2024 [9]	2	3	push-through technique with blunt tipped spatula	12% C3F8	1	1.75 +- 0.35	1.0 +- 0.99	886.5 +- 268.02	620.5 +- 176	30.0%
Daphna et al. 2025 [8]	24	12	push-through technique with spatula or injector	Air, 20% SF6, 10% C3F8	1	1.88 +- 0.79 (n=15)	1.34 +- 0.57 (n=15)	759 +- 116 (n=17)	613 +- 135 (n=17)	19.2%
Lapid-Gortzak and Van Der Meulen 2025 [12]	3*	6	push-through technique with cartridge and forceps	N/A	3.3	2.28 +- 0.17	1.33 +- 0.77	925.33 +- 343.5	760.33 +- 434.03	17.8%
Donner et al. 2025 [21]	11	3	-	10% C3F8	1	-	Descriptive only†	686.27 +- 150.83	456.6 +- 62.72	33.5%
Fontana et al. 2025 [5]	7	24	push-through technique using a blunt spatula	10% C3F8	1.57	1.32 +- 0.23	0.95 +- 0.28	805 +- 131	577 +- 90	28.3%

Surgical Technique and Primary Tamponade Choice

The most commonly used surgical approach was the push-through insertion technique with a blunt spatula, reported in most case series. Several studies utilised pull-through techniques, including suture-assisted or glide-assisted insertion methods.

Primary tamponade selection varied across studies and included 10% or 12% C3F8, 20% SF6, and air. Among these, C3F8 was the most frequently used tamponade. The use of anchoring sutures varied, with means ranging from zero to three sutures depending on surgeon preference and case complexity. 

Where specified, rebubbling procedures were performed using air, 10% C3F8, or 20% SF6. Several reports did not detail the exact tamponade used. In all cases where rebubbling was described, the intervention involved intraocular gas tamponade intended to promote reattachment of the implant.

Complications of EndoArt

Table 6 lists the complications of EndoArt for the 12 included studies.

**Table 6:** Complications of EndoArt *seven patients were excluded due to an overlapping cohort from a previous study. IOL: Intraocular lens; IOP: Intraocular pressure; CCT: Central corneal thickness

Study	Eyes (n)	Eyes with >= 1 rebubbling n/N (%)	Total Rebubbling procedures	Other Complications
Auffarth et al. 2021 [6]	2	2/2 (100%)	2	-
Abusayf et al. 2023 [16]	1	1/1 (100%)	1	loss of first EndoArt to vitreous, IOL subluxation
Rens et al. 2024 [17]	1	0	0	-
Reddy et al. 2024 [18]	1	0	0	-
Wiedemann et al. 2024 [7]	14	8/14 (57%)	13	IOP rise (n=3), persistent high CCT (n=1)
Vishwakarma et al. 2024 [19]	1	1/1 (100%)	3	-
Kobayashi et al. 2024 [20]	1	1/1 (100%)	1	-
Romano et al. 2024 [9]	2	0	0	-
Daphna et al. 2025 [8]	24	18/22 (82%)	-	eye pain (n=6), raised IOP (n=4), Conjunctivitis (n=2), implant explanted due to insufficient attachment (n=5), subepithelial fibrosis (n=1)
Lapid-Gortzak and Van Der Meulen 2025 [12]	3*	3/3 (100%)	6	-
Donner et al. 2025 [21]	11	4/11 (36%)	7	microbial keratitis (n=3)
Fontana et al. 2025 [5]	7	4/7 (57%)	6	-

Results of Meta-Analysis

**Figure 2:**
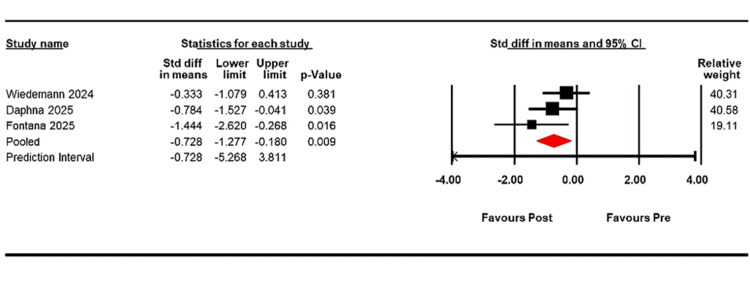
Forest plot showing the change in VA following EndoArt implantation. Data is presented as the SMD with 95% CI VA: visual acuity; SMD: standard mean difference; CI: Confidence Interval

In Figure 2, the change in VA was assessed across three studies, revealing a standard mean difference of -0.728 with a CI of -1.277 to -0.180, indicating a statistically significant difference (p = 0.009). The mean post-treatment VA was 1.212 logMAR (CI, 0.910 to 1.515; p < 0.001), compared with a mean pre-treatment VA of 1.561 (CI, 1.239 to 1.883; p < 0.001). Heterogeneity was low (Q = 2.51, df = 2, I2 = 20.428%; p = 0.285).

**Figure 3:**
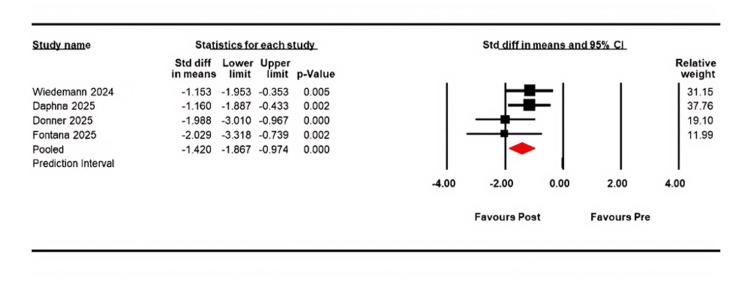
Forest plot showing the change in CCT after EndoArt implantation. Data is presented as the SMD with 95% CI CCT: central corneal thickness; SMD: standard mean difference; CI: confidence interval

In Figure 3, the change in CCT was evaluated across four studies, with a standard mean difference of -1.420 and a CI of -1.867 to -0.974, demonstrating a highly statistically significant difference (p < 0.001). The mean post-treatment CCT was 554.07 μm (CI, 467.85 to 640.29; p < 0.001), compared with a pre-treatment mean of 755.46 (CI, 714.11 to 796.81; p < 0.001). Heterogeneity was low (Q = 2.96, df = 3, I² = 0%; p = 0.397).

**Figure 4:**
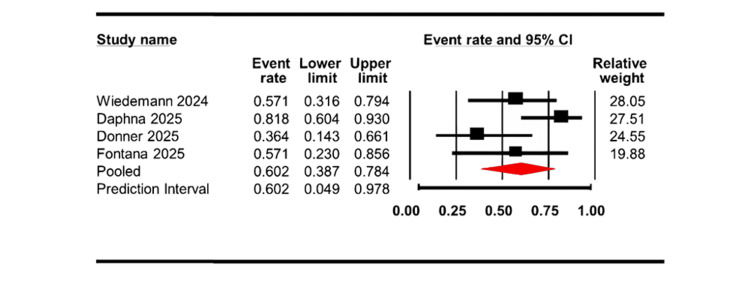
Forest plot of pooled rebubbling rate following EndoArt implantation. Data is presented as event rates with 95% CI CI: confidence interval

In Figure 4, the rebubbling rate was evaluated across four studies, yielding a pooled event rate of 0.602 with a CI of 0.387 to 0.784. The analysis indicated a moderate level of heterogeneity (Q = 6.37, df = 3, I² = 52.88%; p = 0.095).

Discussion

The development of EndoArt, an artificial corneal endothelial implant, represents a novel therapeutic alternative to donor-derived EK for chronic corneal oedema. This systematic review synthesises the clinical outcomes, safety profile, and complications reported in the 12 studies published between 2020 and 2025. Across these studies, EndoArt demonstrated significant improvements in VA and CCT, with pooled analyses confirming statistically significant benefits in both parameters. However, relatively high rebubbling rates highlight the need for cautious interpretation of these findings.

Clinical Outcomes

Across included studies, EndoArt implantation resulted in substantial reductions in CCT, reflecting effective deturgescence of oedematous corneas. This finding is consistent with an early case series by Lapid-Gortzak et al. (2025), who demonstrated rapid stromal clearing following EndoArt implantation in eyes with pseudophakic bullous keratopathy (PBK) [12]. Some studies noted partial recurrence of oedema, most often in eyes requiring rebubbling, highlighting the importance of stable implant adherence for long-term efficacy.

VA outcomes, though significantly improved, remained modest in absolute terms, with a pooled mean postoperative VA of 1.21 logMAR. This limited gain likely reflects the characteristics of the included cohorts, many of which have coexisting ocular comorbidities such as glaucoma, prior failed grafts, and retinal disease. Eyes with greater visual potential may achieve better functional outcomes, but such cases are currently under-represented in the published literature.

Complications and Safety Profile

Implant detachment requiring rebubbling was the most common complication, with a pooled rebubbling rate of 60%, substantially higher than reported for DMEK (3%) [1]. Variability in tamponade choice (air vs SF₆ vs C₃F₈) and the selective use of anchoring sutures may partly explain differences across studies. Anchoring sutures, shown by Fontana et al. (2025) to reduce early dislocation, may also introduce focal interface opacity or trauma [5].

Other complications reported within the included studies were relatively infrequent and included interface haze, isolated cases of implant opacification, and transient intraocular pressure elevation. No cases of immune rejection were reported, reflecting a theoretical advantage of a synthetic implant.

Comparison With Donor-Derived Endothelial Keratoplasty

Donor-derived endothelial keratoplasty, particularly DMEK and DSAEK, remains the gold standard for endothelial failure. However, the global shortage of donor corneas limits access, particularly in low- and middle-income countries [3]. EndoArt offers a scalable, off-the-shelf alternative that could potentially expand treatment access. Nevertheless, functional outcomes are currently inferior to DMEK, and complication rates are higher.

The present review suggests that EndoArt should currently be viewed as a complementary option rather than a substitute for donor EK. It provides a viable alternative for patients at high immunological risk, those with multiple prior graft failures, or those without access to donor tissue.

Patient Selection

The studies included in this review highlight the importance of careful patient selection. Most cases involved elderly patients with pseudophakic bullous keratopathy, prior graft failure, or contraindications to repeat keratoplasty. In such eyes, EndoArt appears to provide meaningful symptomatic relief, such as reduction in pain and photophobia and improved corneal clarity, even when VA improvements are limited. Lapid-Gortzak et al. (2025) noted that subjective patient satisfaction, particularly reduction in photophobia and tearing, was often disproportionately higher than objective VA improvement [12]. This suggests that EndoArt may have a valuable role in improving the quality of life in otherwise refractory patients.

Limitations of Current Evidence

Several limitations of the evidence base must be acknowledged. First, the sample sizes across included studies remain small, with most reporting fewer than 20 eyes. The largest cohort to date, reported by Daphna et al. (2025), included 24 eyes but with 17 patients completing 12 months of follow-up [8]. Second, the absence of randomised controlled trials limits the strength of evidence, and most available data is derived from case series with inherent risks of bias. Third, outcome reporting lacks uniformity, particularly regarding the definition of implant success, making pooled synthesis challenging.

Another critical limitation is the short follow-up duration. Most studies reported outcomes up to 12 months, with only a minority of eyes extending up to 24 months. Given the synthetic nature of the implant, long-term biocompatibility, durability, and risk of late complications such as calcification or biofilm formation remain unknown. The impact of repeated re-bubbling procedures also remains unknown.

A further limitation is that the review was not registered in PROSPERO prior to study selection.

Future Directions

Future research should focus on three areas: optimisation of surgical technique, long-term safety studies, and direct comparative trials with donor-derived EK. Technical refinements, such as bioengineered surface modifications to improve stromal adhesion or the standardisation of tamponade and suturing strategies, may reduce detachment rates. 

Equally important is the need for studies with larger sample sizes. The current literature is largely composed of small case series, which limits statistical power, precludes robust subgroup analyses (e.g., phakic versus pseudophakic eyes), and introduces risk of selection bias. Until such data is available, its use should remain reserved for patients with limited alternatives or as a bridge therapy where donor tissue is unavailable.

## Conclusions

This systematic review demonstrates that EndoArt implantation provides significant improvement in CCT and modest improvement in VA for patients with chronic corneal oedema secondary to endothelial dysfunction. Its safety profile appears favourable with respect to immunological complications, but high rates of detachment and rebubbling will likely remain major barriers to wider adoption. Compared with donor-derived endothelial keratoplasty, reported functional outcomes to date remain inferior, although off-the-shelf availability, and lack of dependence on donor tissue, and absence of immune rejection confer important advantages that may be particularly relevant for patients who are poor candidates for donor keratoplasty or in healthcare systems facing donor shortages. Nevertheless, current evidence is limited by small sample sizes, heterogeneity, and short follow-up. Larger, well-designed clinical trials are required to determine comparative efficacy against DMEK and DSAEK. At present, EndoArt should be considered a salvage therapy for selected patients who are poor candidates for donor keratoplasty.
